# Modified systemic immune-inflammatory index, modified systemic inflammatory response index and hemoglobin-albumin-lymphocyte-platelet score may serve as markers for evaluating the efficacy of neoadjuvant therapy in breast cancer patients

**DOI:** 10.3389/fonc.2026.1746164

**Published:** 2026-02-13

**Authors:** Yuhong Gan, Rongkun Zhu, Jinyuan Li, Qiuming Wang, Xiaobin Meng

**Affiliations:** 1 Department of Clinical Pharmacy, Meizhou People’s Hospital, Meizhou Academy of Medical Sciences, Meizhou, China; 2 Pelvic Radiotherapy Department, Meizhou People’s Hospital, Meizhou Academy of Medical Sciences, Meizhou, China; 3 Department of Medical Oncology, Meizhou People’s Hospital, Meizhou Academy of Medical Sciences, Meizhou, China

**Keywords:** breast cancer, hemoglobin-albumin-lymphocyte-platelet score, modified systemic immune-inflammatory index, modified systemic inflammatory response index, neoadjuvant therapy

## Abstract

**Objective:**

To explore the evaluation value of the modified systemic immune-inflammatory index (mSII), modified systemic inflammatory response index (mSIRI) and hemoglobin-albumin-lymphocyte-platelet (HALP) score for the efficacy of neoadjuvant therapy (NAT) in breast cancer.

**Methods:**

A total of 343 breast cancer patients who received NAT at Meizhou People’s Hospital from June 2016 to October 2023 were analyzed. Clinical and pathological data before treatment and peripheral blood detection indicators were collected. A response to NAT was defined as the achievement of pathological complete response (CR) or partial response (PR). mSII, mSIRI, and HALP score were calculated, and the receiver operating characteristic (ROC) curve analysis was used to evaluate the predictive efficacy of each indicator. The relationship between NAT efficacy and mSII, mSIRI, HALP was analyzed.

**Results:**

179 patients (52.2%) showed no response to NAT, while 164 (47.8%) exhibited a response. The NAT-responsive group had a higher proportion of HER2-positive and TNBC subtypes compared with the nonresponsive group (*p* = 0.007). Compared with the NAT-nonresponsive group, the NAT-responsive group had significantly higher levels of mSII (*p* < 0.001) and mSIRI (*p* < 0.001), while the HALP level was notably lower (*p* = 0.001). The cutoff value of mSII was 2500.145 (area under the ROC curve [AUC]=0.676), while mSIRI had a cutoff of 680.92 (AUC = 0.679) and HALP had a cutoff of 33.385 (AUC = 0.600) by ROC curve analysis. Logistic regression analysis demonstrated that non-luminal subtype (odds ratio [OR]=2.059, 95% confidence interval [CI]=1.294–3.277, *p* = 0.002), elevated mSII (OR = 3.665, 95% CI = 2.235–6.010, *p* < 0.001), increased mSIRI (OR = 3.860, 95% CI = 2.350–6.338, *p* < 0.001), and reduced HALP (OR = 2.267, 95% CI = 1.411–3.643, *p* = 0.001) were independently associated with NAT efficacy.

**Conclusions:**

Elevated mSII, mSIRI, and low HALP score were associated with the effectiveness of NAT in breast cancer. mSII, mSIRI, and HALP score may serve as valuable predictive indicators for the effectiveness of neoadjuvant therapy in breast cancer.

## Introduction

Breast cancer is a malignant tumor that originates from the epithelial cells of the mammary ducts or the lobules (1). The latest statistics show that breast cancer has become the second most common malignant tumor worldwide, and it is the most lethal malignant tumor for women (2). Based on the expression levels of hormone receptors (HR) — which include estrogen receptor (ER) and progesterone receptor (PR), human epidermal growth factor receptor 2 (HER2), and Ki67 (a proliferation marker), breast cancer is categorized into four molecular subtypes: luminal A, luminal B, HER2-enriched (HER2+), and triple-negative breast cancer (TNBC) (3). Breast cancer is a highly heterogeneous malignant tumor, showing significant differences in terms of histological type, molecular classification, and clinical prognosis.

Neoadjuvant therapy (NAT) refers to the systemic treatment administered before local treatment for breast cancer. The core methods include chemotherapy, targeted therapy, endocrine therapy, and immunotherapy, and the treatment plan needs to be tailored based on the patient’s molecular classification, clinical stage, and histological characteristics (4, 5). The aim of NAT is to shrink tumor volume, enhance the feasibility of surgical resection, and raise the likelihood of breast-conserving surgery (6, 7). Additionally, NAT enables the acquisition of data on tumor drug sensitivity, which can guide subsequent treatment strategies and optimize patient prognosis (8). Assessing the response to NAT is crucial for predicting patient survival outcomes and informing follow-up therapeutic plans. The effectiveness of NAT for breast cancer patients is mainly evaluated by the change in tumor diameter as shown in imaging before and after the treatment (9), or by the reduction in tumor cells in the pre-treatment biopsy tissue and surgical specimens based on pathological methods (10, 11). However, the imaging and pathological methods used for evaluating the efficacy of NAT have a lagging nature, and it is difficult to predict the efficacy before the treatment (12, 13). Therefore, it is urgent to find convenient, non-invasive and dynamically monitorable biological assessment indicators to provide a basis for the timely adjustment of the treatment plan.

The inflammatory response in the tumor microenvironment is closely related to the body’s nutritional metabolism status and is a key factor influencing tumor progression and treatment sensitivity (14). Inflammatory cells can accelerate tumor development by promoting angiogenesis and inhibiting anti-tumor immune responses (15), as well as by phagocytosing tumor cells or presenting antigens to activate adaptive immunity (16). Malnutrition weakens the body’s tolerance to treatment and reduces the therapeutic effect (17); excessive nutrition, on the other hand, induces chronic inflammation through metabolic disorders and promotes tumor progression (18, 19). Based on this, some comprehensive indices integrating inflammatory indicators and nutrition-related indicators have demonstrated promising potential in the prognosis assessment of various malignant tumors.

Modified systemic immune-inflammatory index (mSII), modified systemic inflammatory response index (mSIRI), and hemoglobin-albumin-lymphocyte-platelet (HALP) score are all composite indicators focusing on the dimensions of inflammation and nutrition. Their core advantage lies in integrating multiple routine detection indicators through a simple formula to quantitatively reflect the balance of the body’s immune inflammation and the status of nutritional reserves. mSII and mSIRI have optimized the indicator weights based on the basic systemic inflammatory index, making them more in line with clinical practice (20). The HALP score takes into account both nutritional and immune indicators, comprehensively covering the key influencing factors in tumor treatment (21). Some studies have shown that these indicators are related to the diagnosis and prognosis of certain diseases (20, 22–24). However, the specificity, sensitivity and clinical applicability of these indicators in predicting and evaluating the efficacy of NAT in breast cancer have not yet been clearly defined. The main purpose of this study is to address this issue.

## Materials and methods

### Subjects

This study conducted a retrospective analysis on breast cancer patients who received NAT at our hospital from June 2016 to October 2023. Inclusion criteria: (1) Diagnosed with primary breast cancer through pathological tissue examination, and meeting the diagnostic criteria for breast cancer; (2) first received neoadjuvant therapy, with a clear treatment plan and completion of the prescribed course; (3) completed blood routine and biochemical index tests before and after treatment, and had complete clinical data; and (4) the patients and their families gave informed consent and signed the informed consent form. Exclusion criteria: (1) had a history of other malignant tumors or was simultaneously suffering from severe infections, autoimmune diseases, blood system diseases and other diseases that may affect inflammatory and nutritional indicators; (2) received anti-inflammatory treatment, immunomodulatory therapy or nutritional support treatment within 1 month before neoadjuvant therapy; (3) patients with incomplete clinical data; and (4) pregnant or lactating women. This study was supported by the Ethics Committee of the Meizhou People’s Hospital.

### Neoadjuvant treatment regimens and efficacy determination

For patients with luminal A, luminal B-like (HER2-negative), and TNBC, the TEC regimen was administered: docetaxel (T) at 75mg/m^2^ or albumin-bound paclitaxel at 260mg/m^2^, plus epirubicin (E) at 80mg/m^2^ and cyclophosphamide (C) at 500mg/m^2^. Meanwhile, those diagnosed with luminal B-like (HER2-positive) and HER2-positive subtype breast cancer received either the TCbHP or TCbH regimen, consisting of docetaxel (T) 75mg/m^2^ or albumin-bound paclitaxel 260mg/m^2^, carboplatin (Cb) with an area under curve (AUC) of 6, trastuzumab (H) (8mg/kg as the loading dose and 6mg/kg for subsequent doses), and pertuzumab (840mg as the initial dose followed by 420mg thereafter). A single treatment cycle lasts 21 days. Following the completion of 6 treatment cycles, patients underwent evaluation of NAT efficacy prior to undergoing surgery.

The efficacy of NAT is classified into four grades: complete response (CR), partial response (PR), stable disease (SD), and progressive disease (PD). Specifically, CR indicates that all target lesions have disappeared and no new lesions have emerged. The pathological examination shows that there are no cancer cells remaining in the primary tumor and regional lymph nodes. PR indicates that the sum of the maximum diameters of the target lesions has decreased by ≥30% compared to before treatment. SD indicates that the sum of the maximum diameters of the target lesions has decreased by <30% or increased by <20% compared to before treatment. PD indicates that the sum of the maximum diameters of the target lesions has increased by ≥20% compared to before treatment or new lesions have appeared. The CR + PR is defined as NAT-responsive group, and SD + PD treatment ineffective group is defined as NAT-nonresponsive group.

### Data collection

Clinicopathological characteristics of the patients were collected, encompassing gender, age, body mass index (BMI), hypertension, diabetes mellitus, family history of cancer, TNM stage, and molecular subtypes of breast cancer. BMI was divided into three grades: underweight (<18.5 kg/m^2^), normal weight (18.5-23.9 kg/m^2^), and overweight (≥24.0 kg/m^2^) (25, 26). The results of platelet count, neutrophil count, lymphocyte count, hemoglobin, and serum albumin of the patients before NAT were collected.

### Data processing and statistical analysis

The comprehensive indices mSII, mSIRI, and HALP were calculated according to the following formulas:


mSII=platelet×neutrophil/lymphocyte×ln(albumin);

mSIRI=platelet×neutrophil/lymphocyte;

HALP score is based on hemoglobin (g/L), serum albumin (g/L), lymphocyte count (×10^9^/L) and platelet count (×10^9^/L), and the calculation formula is: HALP = hemoglobin × albumin × lymphocyte count/platelet count.

Continuous variables following a normal distribution were presented as mean ± standard deviation, while those deviating from normal distribution were described as median (25th percentile, 75th percentile). Count data were expressed as case number (%). Receiver operating characteristic (ROC) curve analysis was employed to identify the optimal cutoff values of mSII, mSIRI, and HALP for differentiating the efficacy of NAT. The discriminative ability of mSII, mSIRI, and HALP in distinguishing NAT-responsive from NAT-nonresponsive cases was assessed by computing the area under the receiver operating characteristic (ROC) curve (AUC), with the optimal cut-off values of these three indices identified via the Youden index. The Chi-square test or Fisher’s exact test was used to assess the association between NAT efficacy and clinicopathological characteristics of breast cancer patients. The relationship between NAT efficacy and mSII, mSIRI, HALP was evaluated via nonparametric test. The multiple comparisons of the association between clinicopathological features and NAT efficacy were corrected using the Bonferroni correction method. Logistic regression analysis was applied to explore the correlations of these inflammatory markers and clinicopathological features with NAT efficacy in breast cancer patients. A two-tailed *p* value <0.05 was considered statistically significant. All data analyses were performed using SPSS statistical software version 26.0 (IBM Inc., USA).

## Results

### Clinicopathological features of breast cancer patients received NAT

Of the total cases, 147 (42.9%) were aged < 55 years and 196 (57.1%) were aged ≥ 55 years. Regarding body weight status, 13 cases (3.8%) were underweight and 171 cases (49.9%) were overweight. The proportions of patients with hypertension, diabetes mellitus, and a family history of cancer were 47 (13.7%), 27 (7.9%), and 27 (7.9%), respectively. For T stage distribution, there were 9 (2.6%), 165 (48.1%), 109 (31.8%), and 58 (16.9%) patients in stages T1, T2, T3, and T4, respectively. As for N stage, 18 (5.2%), 120 (35.0%), 81 (23.6%), and 124 (36.2%) patients were categorized into stages N0, N1, N2, and N3, respectively. In terms of molecular subtypes, the numbers of patients with luminal A, luminal B, HER2+, and TNBC were 16 (4.7%), 177 (51.6%), 77 (22.4%), and 73 (21.3%), respectively. The levels of mSII, mSIRI, and HALP in these breast cancer patients were 2254.4 (1561.5, 3251.7), 601.3 (411.0, 876.4), and 35.4 (26.7, 47.2), respectively (**Table 1**).

**Table 1:** The clinicopathological features of breast cancer patients received NAT.

Clinicopathological features	Breast cancer patients (n=343)
Age (Years)	
<50, n (%)	147 (42.9%)
≥50, n (%)	196 (57.1%)
BMI (kg/m^2^)	
Underweight, n (%)	13 (3.8%)
Normal weight, n (%)	134 (39.1%)
Overweight, n (%)	171 (49.9%)
Unknown, n (%)	25 (7.3%)
Hypertension	
No, n (%)	296 (86.3%)
Yes, n (%)	47 (13.7%)
Diabetes mellitus	
No, n (%)	316 (92.1%)
Yes, n (%)	27 (7.9%)
Family history of cancer	
No, n (%)	316 (92.1%)
Yes, n (%)	27 (7.9%)
T stage	
T1, n (%)	9 (2.6%)
T2, n (%)	165 (48.1%)
T3, n (%)	109 (31.8%)
T4, n (%)	58 (16.9%)
Tx, n (%)	2 (0.6%)
N stage	
N0, n (%)	18 (5.2%)
N1, n (%)	120 (35.0%)
N2, n (%)	81 (23.6%)
N3, n (%)	124 (36.2%)
Molecular subtypes	
Luminal A, n (%)	16 (4.7%)
Luminal B, n (%)	177 (51.6%)
HER2+, n (%)	77 (22.4%)
TNBC, n (%)	73 (21.3%)
Inflammation and nutritional status indices	
mSII, median (IQR)	2254.4 (1561.5, 3251.7)
mSIRI, median (IQR)	601.3 (411.0, 876.4)
HALP, median (IQR)	35.4 (26.7, 47.2)

HER2, human epidermal growth factor receptor 2; TNBC, triple negative breast cancer; mSII, modified systemic immune-inflammatory index; mSIRI, modified systemic inflammatory response index; HALP, hemoglobin-albumin-lymphocyte-platelet score; IQR, interquartile range.

### Comparison of clinicopathological features among between NAT-nonresponsive group and NAT-responsive group

In the present study, 179 patients (52.2%) showed no response to NAT, while 164 (47.8%) exhibited a response. The NAT-responsive group had a higher proportion of HER2-positive and TNBC subtypes compared with the nonresponsive group (χ^2^ = 11.947, *p* = 0.007). No statistically significant differences were observed between the two groups in terms of hypertension, diabetes mellitus, cancer family history, BMI distribution, or disease stages (all *p*>0.05) (**Table 2**).

**Table 2:** Comparison of clinicopathological features among between NAT-nonresponsive group and NAT-responsive group.

Clinicopathological features	NAT-nonresponsive group (n=179)	NAT-responsive group (n=164)	*p* values
Age (Years)			
<50, n (%)	73(40.8%)	74(45.1%)	0.445 (χ^2^ = 0.658)
≥50, n (%)	106(59.2%)	90(54.9%)	
BMI (kg/m^2^)			
Underweight, n (%)	7(3.9%)	6(3.7%)	0.867 (χ^2^ = 0.332)
Normal weight, n (%)	67(37.4%)	67(40.9%)	
Overweight, n (%)	91(50.8%)	80(48.8%)	
Hypertension			
No, n (%)	153(85.5%)	143(87.2%)	0.754 (χ^2^ = 0.214)
Yes, n (%)	26(14.5%)	21(12.8%)	
Diabetes mellitus			
No, n (%)	163(91.1%)	153(93.3%)	0.548 (χ^2^ = 0.588)
Yes, n (%)	16(8.9%)	11(6.7%)	
Family history of cancer			
No, n (%)	164(91.6%)	152(92.7%)	0.841 (χ^2^ = 0.133)
Yes, n (%)	15(8.4%)	12(7.3%)	
T stage			
T1-T2, n (%)	87(48.6%)	87(53.0%)	0.386 (χ^2^ = 0.885)
T3-T4, n (%)	92(51.4%)	75(45.7%)	
N stage			
N0-N1, n (%)	77(43.0%)	61(37.2%)	0.321 (χ^2^ = 1.206)
N2-N3, n (%)	102(57.0%)	103(62.8%)	
Molecular subtypes			
Luminal A, n (%)	9(5.0%)	7(4.3%)	0.007 (χ^2^ = 11.947)
Luminal B, n (%)	107(59.8%)	70(42.7%)	
HER2+, n (%)	35(19.6%)	42(25.6%)	
TNBC, n (%)	28(15.6%)	45(27.4%)	

HER2, human epidermal growth factor receptor 2; TNBC, triple negative breast cancer.

**Figure 1:**
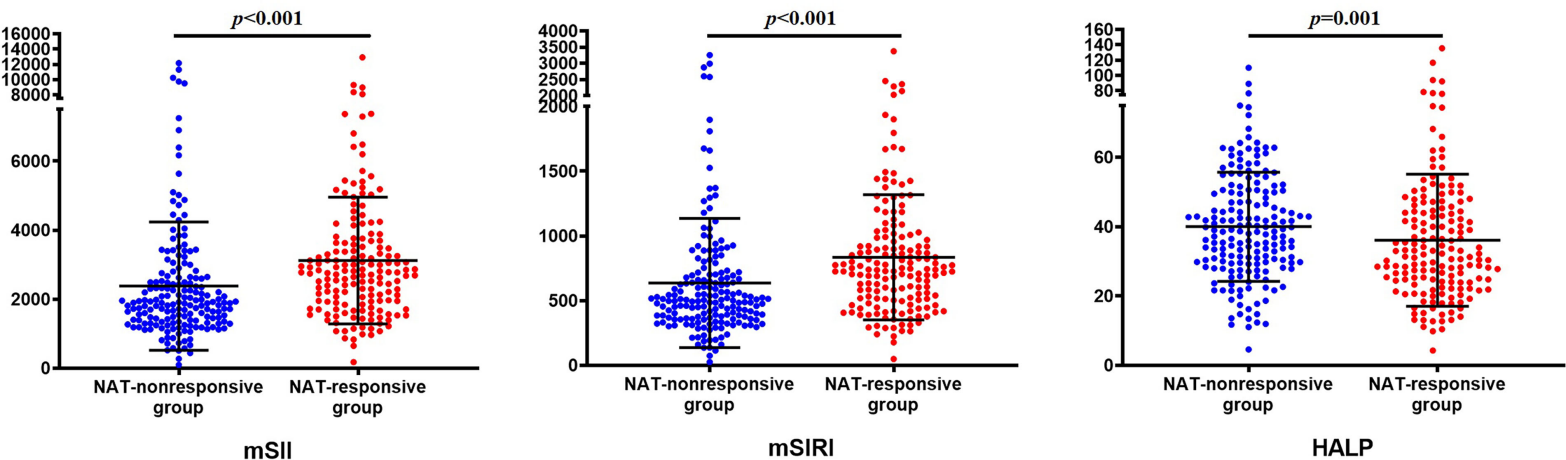
The levels of mSII, mSIRI, and HALP score in patients with or without response to NAT.

Compared with the NAT-nonresponsive group, the NAT-responsive group had significantly higher levels of mSII (2777.1 (1929.3, 3675.1) vs. 1885.4 (1346.0, 2627.0), *p* < 0.001) and mSIRI (739.9 (514.0, 990.6) vs. 505.2 (363.5, 700.7), *p* < 0.001), while the HALP level was notably lower (32.1 (23.9, 44.6) vs. 38.6 (29.6, 50.1), *p* = 0.001) (**Figure 1**).

### Logistic regression analysis of factors associated with the efficacy of NAT

**Figure 2:**
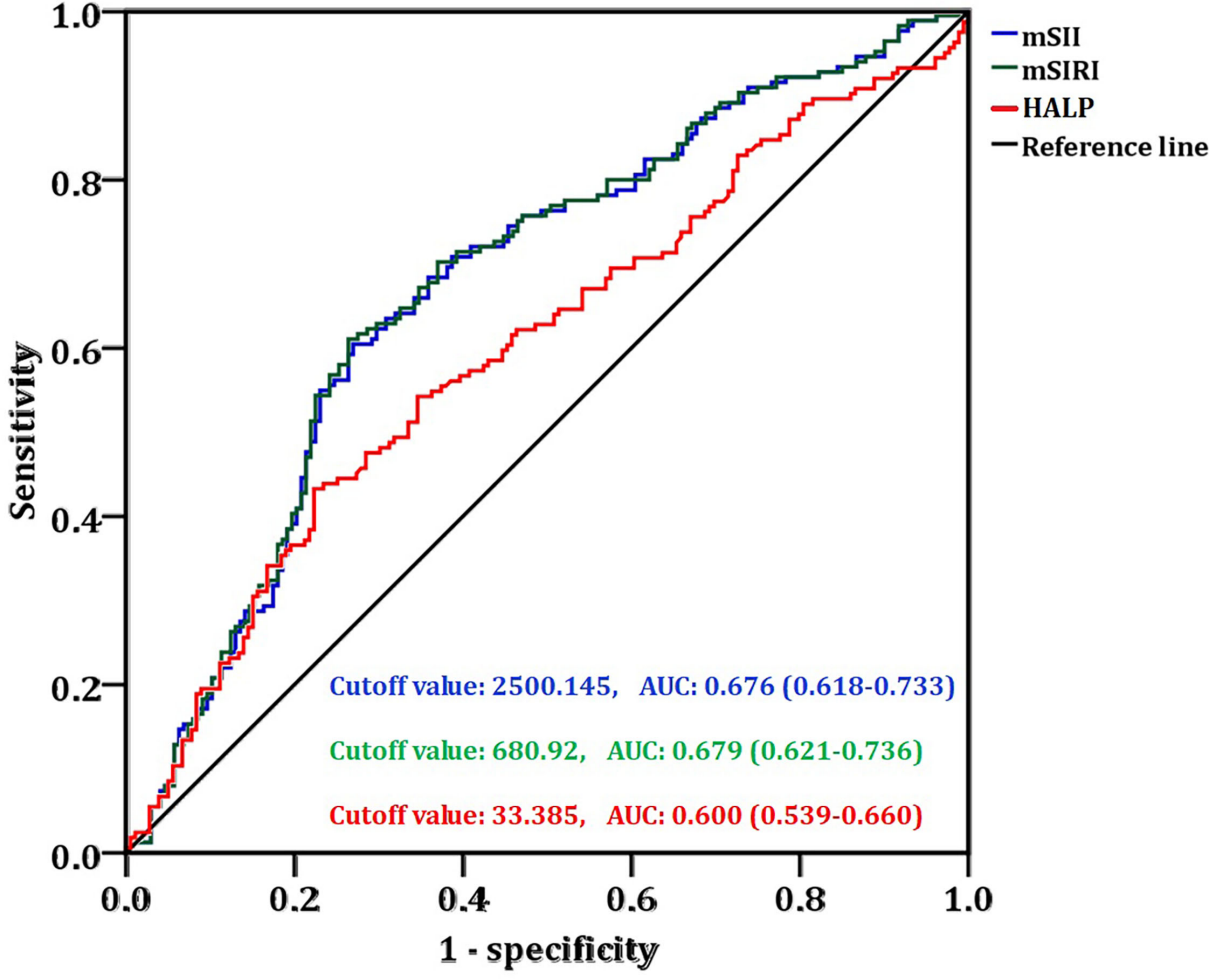
The ROC curve of mSII, mSIRI, and HALP score based on the efficacy of NAT.

ROC curve analysis was performed with NAT efficacy as the endpoint to determine the critical values of mSII, mSIRI, and HALP. The cutoff value of mSII was 2500.145 (sensitivity=60.4%, specificity=73.2%, AUC = 0.676), while mSIRI had a cutoff of 680.92 (sensitivity=61.0%, specificity=73.7%, AUC = 0.679) and HALP had a cutoff of 33.385 (sensitivity=54.3%, specificity=64.8%, AUC = 0.600) (**Figure 2**).

Univariate logistic regression analysis revealed that non-luminal subtype (vs. luminal subtype; odds ratio [OR]=2.080, 95% confidence interval [CI]=1.348-3.210, *p* = 0.001), elevated mSII (≥2500.145 vs. <2500.145; OR = 4.157, 95% CI = 2.637-6.553, *p* < 0.001), increased mSIRI (≥680.92 vs. <680.92; OR = 4.388, 95% CI = 2.777-6.934, *p* < 0.001), and reduced HALP (<33.385 vs. ≥33.385; OR = 2.185, 95% CI = 1.415-3.373, *p* < 0.001) were significantly correlated with NAT efficacy. Multivariate logistic regression analysis demonstrated that non-luminal subtype (OR = 2.059, 95% CI = 1.294-3.277, *p* = 0.002), elevated mSII (OR = 3.665, 95% CI = 2.235-6.010, *p* < 0.001), increased mSIRI (OR = 3.860, 95% CI = 2.350-6.338, *p* < 0.001), and reduced HALP (OR = 2.267, 95% CI = 1.411-3.643, *p* = 0.001) were independently associated with NAT efficacy (**Table 3**).

**Table 3:** Logistic regression analysis of factors associated with the efficacy of NAT in breast cancer patients.

Variables	Crude β/OR (95% CI)	*p* values	Adjusted β/OR (95% CI)	*p* values
T stage (T3-T4 vs. T1-T2)	0.815 (0.533-1.248)	0.347	0.765 (0.485-1.207)	0.250
N stage (N2-N3 vs. N0-N1)	1.275 (0.826-1.966)	0.272	1.160 (0.727-1.850)	0.534
Molecular subtypes (non-luminal vs. luminal)	2.080 (1.348-3.210)	0.001	2.059 (1.294-3.277)	0.002
mSII (≥2500.145 vs. <2500.145)	4.157 (2.637-6.553)	<0.001	3.665 (2.235-6.010)	<0.001
mSIRI (≥680.92 vs. <680.92)	4.388 (2.777-6.934)	<0.001	3.860 (2.350-6.338)	<0.001
HALP (<33.385 vs. ≥33.385)	2.185 (1.415-3.373)	<0.001	2.267 (1.411-3.643)	0.001

OR, odds ratio; CI, confidence interval.

Adjust for: age, gender, BMI, hypertension, diabetes mellitus, and fFamily history of cancer.

### Logistic regression analysis of factors associated with the efficacy of NAT in HER2+ and TNBC patients, respectively

In patients with HER2+, multivariate logistic regression analysis showed that elevated mSII (OR = 8.824, 95% CI = 2.851-27.303, *p* < 0.001), increased mSIRI (OR = 8.000, 95% CI = 2.592-24.688, *p* < 0.001) were independently associated with NAT efficacy, whereas no significant correlation was observed between HALP score and NAT efficacy. In patients with TNBC, logistic regression analysis showed that elevated mSII (OR = 4.077, 95% CI = 1.085-15.314, *p* = 0.037), increased mSIRI (OR = 5.319, 95% CI = 1.384-20.442, *p=*0.015), and reduced HALP (OR = 10.791, 95% CI = 2.366-49.221, *p* = 0.002) were independently associated with NAT efficacy (**Table 4**). The results showed that both mSII and mSIRI were correlated with the efficacy of NAT in patients with HER2+ and TNBC subtypes; HALP was associated with NAT efficacy in patients with the TNBC subtype, but not in those with the HER2+ subtype.

**Table 4:** Logistic regression analysis of factors associated with the efficacy of NAT in HER2+ and TNBC patients, respectively.

Variables	HER2+ patients				TNBC patients			
	Crude β/OR (95% CI)	*p* values	Adjusted β/OR (95% CI)	*p* values	Crude β/OR (95% CI)	*p* values	Adjusted β/OR (95% CI)	*p* values
T stage (T3-T4 vs. T1-T2)	1.123 (0.455-2.770)	0.802	1.008 (0.338-3.012)	0.988	0.872 (0.332-2.291)	0.781	0.649 (0.203-2.069)	0.464
N stage (N2-N3 vs. N0-N1)	0.692 (0.256-1.869)	0.468	0.854 (0.263-2.768)	0.792	0.780 (0.288-2.112)	0.625	0.421 (0.119-1.489)	0.179
mSII (≥2500.145 vs. <2500.145)	8.824 (2.851-27.303)	<0.001	7.421 (2.220-24.808)	0.001	2.889 (1.074-7.770)	0.036	4.077 (1.085-15.314)	0.037
mSIRI (≥680.92 vs. <680.92)	8.000 (2.592-24.688)	<0.001	6.465 (1.966-21.259)	0.002	3.477 (1.284-9.415)	0.014	5.319 (1.384-20.442)	0.015
HALP (<33.385 vs. ≥33.385)	2.273 (0.878-5.883)	0.091	2.283 (0.789-6.601)	0.128	3.421 (1.245-9.403)	0.017	10.791 (2.366-49.221)	0.002

OR, odds ratio; CI, confidence interval.

Adjust for: age, gender, BMI, hypertension, diabetes mellitus, and family history of cancer.

## Discussion

This study focused on the mSII, mSIRI, and HALP score, systematically exploring their value in evaluating the efficacy of NAT in breast cancer. The results showed that the levels of mSII and mSIRI in the NAT-responsive group were significantly higher than those in the NAT-nonresponsive group, while the HALP score was significantly lower than that in the ineffective group. mSII, mSIRI, and HALP score were significantly associated with the NAT efficacy. It suggests that high levels of inflammatory-related indices and low levels of the nutritional-immune composite score can serve as potential markers for NAT efficacy in breast cancer.

The clinical significance of this research result mainly lies in the following aspects. Firstly, the prediction is convenient and has wide applicability. The three indicators are all calculated based on the blood routine and biochemical test results before treatment. No additional invasive biopsy or special molecular testing is required. The detection cost is low and the operation is simple, making it easy to be promoted and applied in primary medical institutions. It can be used as a routine efficacy screening indicator before neoadjuvant treatment. Secondly, it achieves the clinical goal of early prediction and early intervention. The efficacy prediction can be completed at the baseline level before treatment. For those predicted to be effective, the confidence in the original treatment plan can be strengthened. For those predicted to have a higher risk of ineffectiveness, a multidisciplinary consultation can be initiated in advance to promptly adjust the treatment plan, avoiding adverse reactions caused by ineffective treatment.

The value of the comprehensive indices integrating inflammatory indicators and nutrition-related indicators in breast cancer has been studied in some previous studies. Guo et al. found that the high pre-treatment systemic immune inflammatory response index (SII) was associated with shorter overall survival (OS) in TNBC patients (27). Li et al. found that the elevated systemic inflammatory response index (SIRI) before treatment was an independent risk factor for the low disease-free survival (DFS) rate of breast cancer patients after surgery (28). Yamanouchi K et al. revealed that stage IV breast cancer patients with a low level of SIRI had significantly better OS rates than those with a high level of SIRI (29). The research conducted by Xiong et al. suggested that a high SIRI level may be associated with lymph node metastasis in breast cancer patients (30). Zhang et al. found that elevated SIRI may be associated with the efficacy of NAT in breast cancer patients, but not SII (31). In addition, some studies revealed that there is no relationship between the systemic inflammatory response indicators SII and SIRI and the effectiveness of NAT (32), OS (33) in breast cancer.

In this study, high mSII and mSIRI may be independent factors associated with the effectiveness of NAT in breast cancer. The mechanism by which high levels of mSII and mSIRI predict effective treatment essentially reflects the pathological and physiological manifestation of the coordinated regulation of tumor treatment sensitivity through the activation of inflammation in the tumor microenvironment, the immune response, and nutritional metabolism. High levels of neutrophils can degrade the extracellular matrix of tumor cells and promote angiogenesis by releasing mediators such as neutrophil elastase and matrix metalloproteinase-9 (MMP-9), creating favorable conditions for drug penetration (34, 35). At the same time, moderately elevated platelets can enhance the sensitivity of tumor cells to chemotherapy drugs by releasing cytokines such as platelet-derived growth factor (36, 37). When the lymphocyte count is relatively stable, this “pro-inflammatory effect” may more easily induce tumor cell apoptosis, which may stem from the special mechanism of “inflammation-mediated drug sensitivity” in the neoadjuvant treatment scenario (38, 39). Moreover, as the core effector cell of anti-tumor immunity, when the number of lymphocytes is maintained at a certain level, it can enhance the anti-tumor immune response induced by treatment by recognizing tumor antigens (40). The results of this study have further expanded the application scope of systemic inflammatory indicators in the field of breast cancer treatment. However, the value of systemic inflammatory indicators in the assessment of NAT efficacy remains controversial. Future studies could further explore the value of the combined application of the aforementioned indicators with imaging parameters (such as tumor regression rate on breast MRI), pathological complete response (pCR) criteria, and molecular subtype markers, and construct multidimensional evaluation models to improve the accuracy and specificity of NAT efficacy prediction.

HALP has been proven to be related to the clinical pathological characteristics and treatment prognosis of various tumors (21). There have also been some studies on its role in breast cancer. Duran A et al. believed that a low HALP score was associated with the aggressiveness of breast cancer (41). The research conducted by Jiang et al. revealed that the preoperative HALP score can serve as a reliable and independent predictor for the OS and progression-free survival (PFS) of breast cancer patients (42). The research by Zhao et al. suggested that the HALP score can serve as a valuable indicator for evaluating the recurrence-free survival (RFS) rate of early-stage breast cancer patients (43). However, some studies found that preoperative HALP score was not associated with the early diagnosis of breast cancer (44), and the effectiveness of neoadjuvant chemotherapy in breast cancer (45, 46).

In this study, HALP may be an independent factor associated with the effectiveness of NAT in breast cancer. A low HALP score predicts effective treatment, reflecting the specific association between tumor metabolism and treatment sensitivity. The HALP score integrates hemoglobin, albumin, lymphocytes, and platelets. Its low level mainly results from moderate reductions in hemoglobin and albumin, which may be related to the nutritional competition effect of tumor cells. During the rapid proliferation of tumor cells, they consume a large amount of nutrients from the body, leading to a mild nutritional depletion before treatment (47, 48). In this mild nutritional depletion state, the metabolic activity of tumor cells is higher (49), and their sensitivity to cytotoxic treatments such as chemotherapy also significantly increases (50). Metabolically active tumor cells are more susceptible to drug-induced DNA damage and apoptosis, while normal cells, with relatively stable metabolism, are more tolerant to treatment (51, 52). At the same time, the mild nutritional depletion state can activate the body’s emergency immune response, promote the infiltration of lymphocytes into the tumor microenvironment, enhance the anti-tumor immune effect, and form a synergistic killing effect with the treatment drugs (53, 54).

In addition, this study found that both the mSII and mSIRI were significantly associated with the efficacy of NAT in patients with HER2+ and TNBC subtypes. These findings suggest that these two inflammation-related indicators may serve as potential common reference markers for evaluating NAT efficacy in the above two breast cancer subtypes. In contrast, the association between the HALP score and NAT efficacy exhibited distinct subtype-specificity: specifically, this score was significantly correlated with NAT efficacy only in TNBC patients, but showed no obvious correlation with that in HER2+ patients. Furthermore, it also reflects that there may be distinct molecular regulatory mechanisms underlying the relationship between the inflammatory microenvironment and treatment response in HER2+ and TNBC subtypes of breast cancer. TNBC is characterized by high heterogeneity and lacks well-defined therapeutic targets (55, 56). Its treatment response is more susceptible to the combined effects of factors such as the body’s nutritional status, immune cell infiltration, and platelet function—factors that are precisely integrated into the HALP score. In contrast, the NAT efficacy of HER2+ breast cancer is more dependent on the specific action of therapeutic drugs on the HER2 target, with nutrition- and immunity-related indicators exerting a relatively lower weight of influence on treatment response (57, 58). This subtype-specific association provides differentiated assessment tools for individualized efficacy prediction in breast cancer of different molecular subtypes, and also offers a direction for further in-depth exploration of the mechanisms underlying treatment responses in distinct breast cancer subtypes.

This study is among the limited number of researches investigating the association between the levels of comprehensive indices (integrating inflammatory and nutrition-related indicators) and NAT efficacy in breast cancer patients. However, this study still has certain limitations. Firstly, this study is a single-center retrospective analysis with a relatively small sample size and potential selection bias. The generalizability of the results requires multi-center, large-sample prospective cohort studies to verify. Secondly, the association between dynamic changes of indicators and therapeutic efficacy was not deeply explored. Only the baseline levels before treatment were analyzed. Future longitudinal studies are needed to monitor the trend of indicator changes after 2 and 4 treatment cycles, and to determine whether the dynamic changes of indicators can further improve the accuracy of efficacy prediction. Thirdly, the patients related to immunotherapy was not included. As immunotherapy is increasingly applied in neoadjuvant treatment, it is necessary to further verify the predictive value of indicators in immunotherapy combination treatment regimens. Finally, the AUC values of mSII, mSIRI, and HALP score for evaluating the NAT efficacy in breast cancer ranged 0.60~0.68, achieving only a moderate level of diagnostic efficacy. When used alone as biomarkers for treatment efficacy assessment, their sensitivity and specificity still have considerable room for improvement. It may stem from the fact that the aforementioned indicators only reflect a single dimension of the body’s systemic inflammatory, immune, or nutritional status, making it difficult to fully encompass the multiple complex factors that influence treatment efficacy, such as tumor heterogeneity, molecular subtypes, and sensitivity to treatment regimens.

## Conclusion

mSII, mSIRI, and HALP score are valuable predictive indicators for the effectiveness of neoadjuvant therapy in breast cancer. The results of this study, which is based on comprehensive indices of non-invasive hematological indicators (mSII, mSIRI, and HALP score) and the therapeutic efficacy of NAT in breast cancer, provide valuable references for the assessment of the therapeutic effect of NAT in breast cancer and the formulation of treatment regimens.

## Data Availability

The original contributions presented in the study are included in the article/supplementary material. Further inquiries can be directed to the corresponding author.
